# Electromechanical Properties of Silver-Plated Yarns and Their Relation to Yarn Construction Parameters

**DOI:** 10.3390/polym15214210

**Published:** 2023-10-24

**Authors:** Johannes Mersch, Hans Winger, Ercan Altinsoy, Chokri Cherif

**Affiliations:** 1 Institute of Textile Machinery and High Performance Materials, TUD Dresden University of Technology, 01069 Dresden, Germany; chokri.cherif@tu-dresden.de; 2 Institute of Measurement Technology, Johannes Kepler University Linz, 4040 Linz, Austria; 3 Institute of Acoustics and Speech Communication, TUD Dresden University of Technology, 01069 Dresden, Germany; hans.winger@tu-dresden.de (H.W.); ercan.altinsoy@tu-dresden.de (E.A.); 4 Centre for Tactile Internet with Human-in-the-Loop (CeTI), TUD Dresden University of Technology, 01069 Dresden, Germany

**Keywords:** electrically conductive yarn, fiber-based sensors, textile strain sensors, silver-plated yarn, electromechanical characterization

## Abstract

For signal transmission and sensing in stretchable structures for human motion monitoring or proprioception of soft robots, textiles with electronically conductive yarns are a promising option. Many recent publications employ silver-plated yarns in knits, braids, wovens for strain or pressure sensing purposes as well as heating fabrics or twisted string actuators. Silver-plated yarns are available in a wide range of base materials, yarn counts and twists. These structural properties significantly influence the electrical and electromechanical behavior of such yarns. However, until now little research has been carried out on the yarns themselves. To close this research gap, several variations of a single yarn type are electromechanically characterized. Additionally, tensile tests with synchronous resistance measurements are performed. From these measurements, sensor metrics are derived and calculated to compare the different variants quantitatively.

## 1. Introduction

With the accelerating, further development of mobile technologies, technical systems are increasingly entering every single area of human life. Whether in medical care, industrial production, education or leisure, modern technologies, e.g., for networking people and machines (human/human; human/machine; machine/machine) are now an essential part of our existence and steadily gaining in importance. As a result of this development, interfaces for the exchange of information between people and technical systems, beyond screen or sound output or classic input options, such as buttons, keys, etc., are becoming more and more the focus of scientific, economic and social interest. A meaningful, safe and not irritating (i.e., intuitive and natural) interaction with technical systems requires intuitively usable input devices and feedback systems.

Since the majority of humankind mostly wears clothing, the approach of integrating such interfaces, i.e., sensor or actuator systems, directly into it seems very promising. Textile-based systems provide a nearly undisturbed wearing of the sensor/actuator-equipped garments. Furthermore, such textile systems can also be used to build fiber composite structures; for example, to produce soft robots.

Since the measurement of strain at the joints of multi-limb systems (e.g., robotic arms or human hands) enables the movement of these limbs relative to each other to be tracked, strain sensors are considered a useful approach to postural movement measurement if conventional external (e.g., radar or optical systems) or internal (usually based on inertial measuring units) tracking systems are required to be avoided. Textile-based transducer systems (especially sensors) and conductors (energy/data) are frequently developed using electrically conductive textile yarns. Among these, silver-coated polyamide yarns are very common. In general, the range of commercially available electrically conductive yarns is increasing more and more. This refers both to the materials used (in addition to those already mentioned, e.g., stainless steel or electrically conductive elastomer fiber yarns are also available) and to different yarn constructions. However, stainless steel fibers possess low stretchability and elastomeric fibers often show non-monotonic strain-resistance behavior [1].

In this paper, only silver-coated PA yarns are discussed. These have similar processing and usage properties as other polymer yarns and can therefore be integrated very well into textile manufacturing processes and products.

The general qualification of such yarns as base materials for the design of fully textile conductor and sensor structures has already been proven and described in detail [2,3,4]. However, if the aim is to go further than the basic proof of concept and develop transducer or transmission systems for specific application scenarios, it is essential to have the most precise knowledge possible of the electromechanical properties of the materials. Transducer and transmission systems have completely opposite objectives in this regard. Conduction systems (→transmission) should show electrical properties that are as constant as possible, independent of external influences, while sensors (→transducer) should have an ideally linear, but always at least unambiguous, relationship between electrical properties and with, at best, a single measurand. In reality, neither is usually the case. Therefore, as already mentioned, it is important to investigate the dependencies between the input parameter in question and the electrical output signal in detail to be able to adapt the entire system to interfering variables.

For a meaningful, i.e., quantitative yarn-based strain measurement, the electromechanical behavior under the tensile load of the potential sensor yarns is the essential parameter set to examine. So far, however, the literature has mainly focused on other external parameters, such as temperature [5,6,7,8], chemicals [9,10,11,12,13] and friction [11,12,14], or used non-cyclic strain tests [4,6,15,16,17], which are only of limited value for characterization of sensors in general and fiber-based sensor materials in particular because they are not able to reproduce transient and settlement phenomena. One specifically relevant example is the study of commercially available yarns in reference [18]. In this study, two of the yarns that will be evaluated in this study were also used. However, the tests were non-cyclical tests with quasi-static strain being measured until break conditions. They found that some of the silver-polyamide yarns had low resistance changes at low strain levels.

Others focused on already processed yarns in the form of braids, knits or wovens [19,20,21]. Therein, the transduction mechanism between strain and resistance change of smart textiles is discussed, particularly for the deformation of loops in a knit. They found that the change is dominated by the resistance change of the yarn itself as well as the length ratios of yarn in the loop. On the other side, contact resistance between yarns in the knit played a minor role. Again, this supports the idea of characterizing the yarns’ electromechanical behavior. In order to select the right material for a specific device or to be able to carry out a targeted (further) development of conductive yarns, a precise investigation of existing materials is very important because the electrical and electromechanical properties of electrically conductive coated yarns are highly dependent on their specific structure, condition and application scenario. Due to the complex interaction of various mechanisms (e.g., material and structural strain, various friction components), the actual yarn behavior cannot be predicted analytically with sufficient precision [22]. By comparing four yarn types based on the same substrate, this paper shows how significant the influence of different finishing steps on the yarn properties is.

## 2. Materials and Methods

Four commercially available yarns from the manufacturers Statex Produktions- und Vertriebs GmbH and Madeira Garnfabrik Rudolf Schmidt KG were used for the tests (see Table 1). All the yarns are based on high tenacity polyamide 6.6 fibers and consist of two twisted threads. A silver coating gives the yarns their good electrical conductivity. All these yarns are regularly utilized in a wide variety of applications.

A special characteristic of these four yarn types and the reason for the choice of these yarns is their close technical relationship. The types HCB, TPU and HC40 are based on S117. They are therefore further developed versions. Like S117, HCB and TPU are manufactured and sold by Statex, but they are equipped with an additional silver layer to increase their electrical conductivity (HC—high conductive). In addition, a protective layer (B—“Beschichtung”, German for coating) is applied to the HC coating, which is based on a silicone oil, to reduce external influences. It is important to note that this coating, in contrast to the TPU coating, does not insulate the yarn.

The TPU yarn is additionally electrically insulated with a thermoplastic polyurethane (TPU) sheath. The construction is thus similar to an insulated cable. The relatively thick TPU cover (92% of the yarn’s weight, 250 µm thickness) is likely to significantly affect the mechanical properties and it severely limits the processability in, for example, knitting or embroidery. Due to the coating, it is likely that the individual filaments are restricted in their relative motion. The lower relative motion could then result in a lower gauge factor. However, the electrical insulation of the conductive yarn to its environment is important for many applications. 

The HC40 yarn, meanwhile, is a further refined version of the HCB. Madeira, as a specialist for sewing and embroidery yarns, achieves a more homogeneous yarn thickness and surface. As far as we were able to investigate, they achieve this by adding another surface coating, which is also not specified for reasons of legal protection; tests, however, point in the direction of a silicone oil coating here too. The performance of Madeira yarns for processing in sewing and embroidery machines is significantly increased compared to the basic materials, which has been achieved by the addition of this silicone oil layer. However, comparative experiments on the processing of different electrically conductive yarns with various textile manufacturing processes that were carried out in our institute showed that the Statex yarns are more suitable for knitting than the specialized Madeira types.

At this point, the effect of the yarn structure on their usability in different manufacturing scenarios becomes clear. In this article, such effects will be demonstrated on the example of their electromechanical properties and also in relation to applications in the field of full-textile transducer and transmission systems.

**Figure 1:**
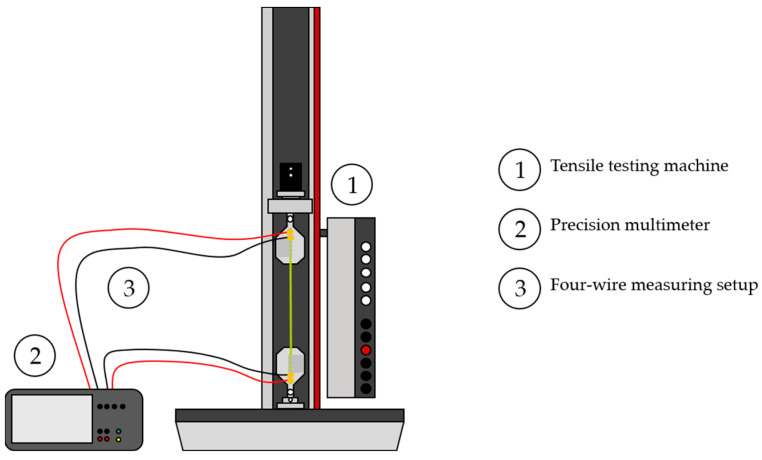
Experimental setup for the determination of the electromechanical properties of electrically conductive yarns (schematic) with current injection through the red wires and voltage sensing over the black wires.

The electromechanical characterization was based on axial tensile tests of the yarns. These were carried out using a standard tensile testing machine of the type zwickiLine Z2.5 (ZwickRoell GmbH & Co. KG, Ulm, Germany) (Figure 1, no. 1). The test is carried out based on the norm DIN EN ISO 2062. In parallel, the electrical resistance of the yarns was measured using a Keithley DAQ6550 precision multimeter (Tektronix Inc., Beaverton, OH, USA) (Figure 1, no. 2). To reduce the effect of wire and contact resistances, a four-wire setup [1] (Figure 1, no. 3) was used to log the electrical resistance.

In order to be able to detect any transient oscillation behavior or similar properties, a multi-cycle test regime is useful and appropriate for the characterization of sensors. Due to the material and structural properties of textile materials and the resulting predisposition to settling phenomena, such repetitive loading and unloading is essential in the characterization of textile-based sensor structures. Ten strain cycles were carried out for each strain level. This was increased in steps of two percent from four to twelve percent or alternately until the yarns broke. The two percent strains are not part of the analyses because a reproducible electromechanical behavior is only given from about four percent strain for the majority of the yarns examined. A higher number of cycles and additional tests with a rest period would improve the significance of the test results, but that would also increase the duration of the tests. The test parameters described in Table 2 were chosen as a reasonable compromise between practicability and the expected quality of the test results. According to the specimen length and testing speed, the strain rate during the tests was 50%/min or 0.83%/s. 

Important properties of strain sensors for human motion monitoring are gauge factor, drift and stiffness, which are each evaluated and then compared. How the parameters are derived is presented during the discussion of the first yarn type. For the evaluation of smart textiles and soft actuators, the conductive yarns are processed on embroidery and knitting machines to be integrated into textile structures. 

## 3. Results and Discussion

In the following section, the results of the electromechanical characterization with the tensile testing machine are presented. Overall, the base resistance, stiffness, gauge factor and linearity of the investigated silver-plated yarns varies significantly.

### 3.1. Characterization of S117 Yarn

**Figure 2:**
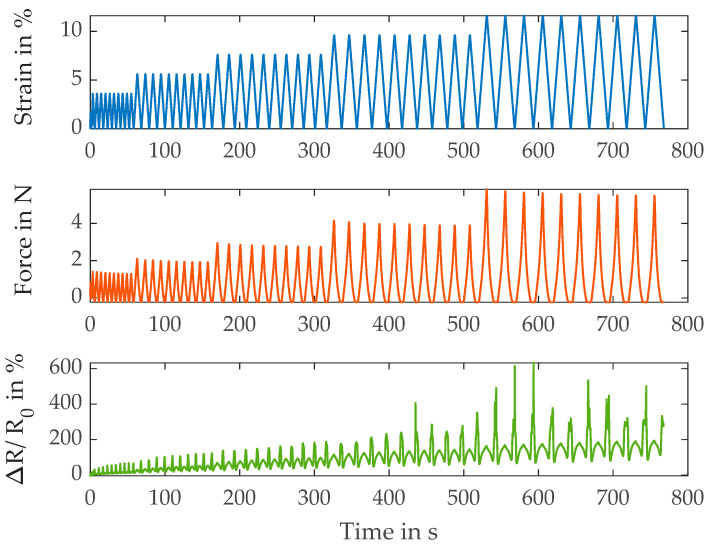
Results of the electromechanical characterization of the S117 yarns.

First, in Figure 2, the relation between strain, force and relative resistance change of the base yarn S117 is shown.

During the loading cycle, the resistance signal coincides well with the strain signal. However, there are large peaks visible at the minimum strain when the yarn should be relaxed. This is an undesired property of the base yarn. Further metrics are listed in Table 3.

The modulus, which in this case is calculated by dividing maximum force of each cycle by strain, rises with the increasing cycle number or strain. This points to the fact that the yarns fibers are straightened first (structural deformation) and move mostly relative to each other before the polyamide material itself is strained (material deformation). In addition, the gauge factor, which is calculated as the resistance change per strain (the relative resistance change at the maximum strain of the respective cycle), rises as well with the increasing strain level. This is likely caused by the described transition between structural and material deformation. At first, the resistance change is mostly dominated by a change of fiber-to-fiber contact points and contact pressure. When the material and thereby the coating is deformed, the resistance change is a higher per percent of strain, leading to a higher gauge factor. In contrast to modulus and gauge factor, the amount of drift decreases at a higher strain. The drift is calculated as the non-recoverable resistance change (the relative resistance change at the end of the cycle at 0% strain) in relation to the overall signal, i.e., maximum resistance change at that strain level. The decrease in the drift at higher strain levels is in agreement with the presented assumption. The resistance change based on fiber-to-fiber contact is based on the friction between fibers. In contrast to the material deformation, where the applied energy is stored and can be recovered when the strain is released, this is not the case for the sliding between the fibers. The energy is dissipated due to friction and cannot be recovered. Since, as has already been explained, the proportion of material strain in the total deformation increases with the rising strain level, which decreases the proportion of frictional losses and thus decreases the signal drift.

### 3.2. Characterization of HCB Yarn

**Figure 3:**
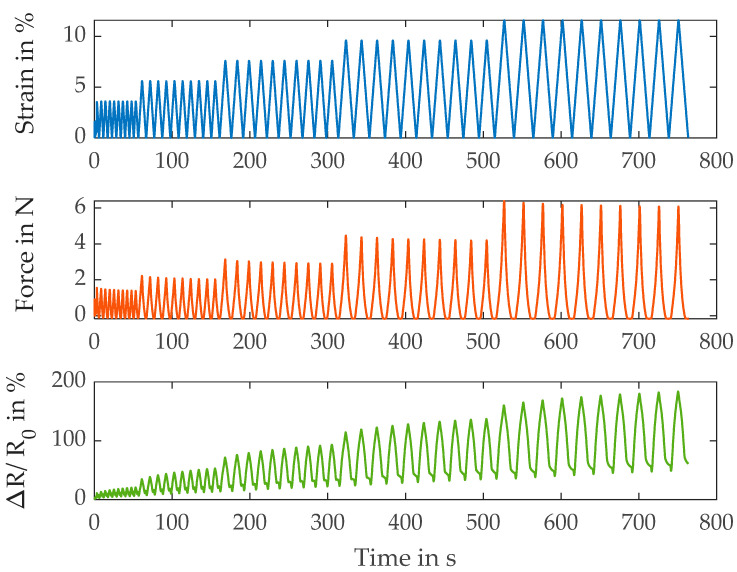
Results of the electromechanical characterization of the S117 HCB yarns.

Figure 3 illustrates the results of the high-conductive (HC) and coated (B) S117 yarn, the HCB. The first difference compared to the non-coated yarn is the absence of secondary peaks at 0% strain. However, the resistance curve differs from the ideal sensor behavior around the minimum strain at each cycle. That is caused by the non-reversible proportion of the strain, which leads to the bending and buckling of the yarn when the strain is taken back. The slightly negative force at each strain minimum supports this observation.

Table 4 lists the calculated metrics for the HCB yarn. The modulus is slightly higher in comparison with the non-coated version (S117), which might be caused by the additional conductive material or by the protective coating. Again, as for the base version, the gauge factor increases with the rising strain but is higher overall. Moreover, the drift is most prominent at low strain levels as is observed in the base S117.

### 3.3. Characterization of HC40 Yarn

**Figure 4:**
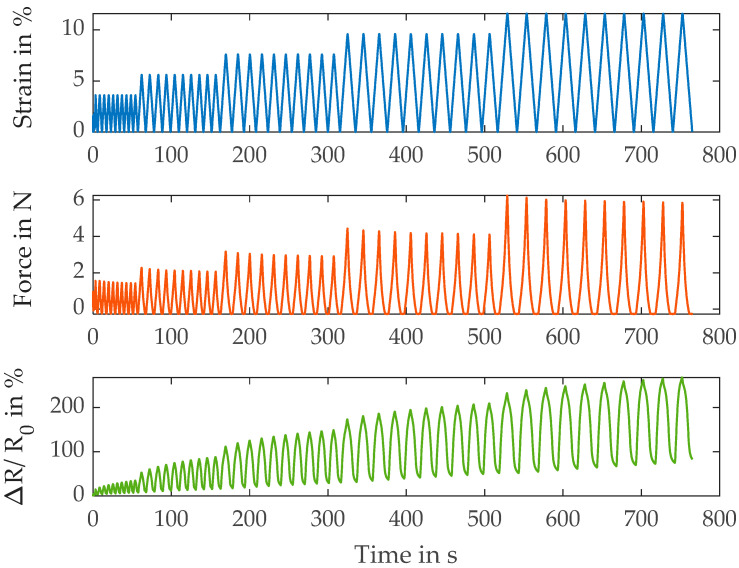
Results of the electromechanical characterization of the Madeira HC40 yarns.

In Figure 4, the results of the HC40 characterization are depicted. In comparison with the first two yarn types, the resistance change is more pronounced overall but the baseline drift is also larger. In addition, the sensor behavior around the strain minimum is improved. Considerable non-linearity is still observed but significantly less in comparison with the S117 versions (S117 and HCB) above.

Table 5 lists the metrics of the Madeira HC40 yarn at the different strain levels. The modulus is nearly identical to the HCB, which suggests that the materials are very similar in their basic setup. This is consistent with the known information on the basic structure of the two types of yarn. As mentioned, the overall resistance change, and thus the gauge factor, is greater than that of the other two versions. Finally, the drift is lowest at the intermediate strain levels; however, the properties are very similar to the HCB type. Consequently, in this case, it is feasible to choose the yarn better suited for the textile process that it is intended for.

### 3.4. Characterization of TPU-Coated Yarn

**Figure 5:**
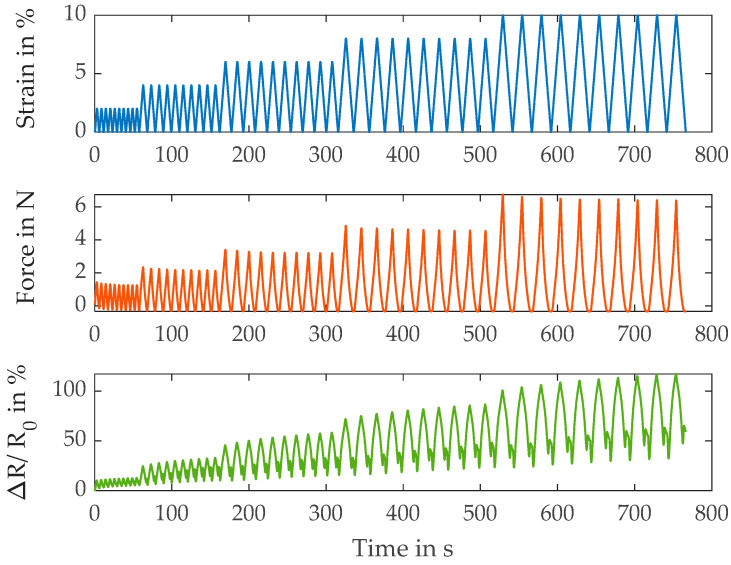
Results of the electromechanical characterization of the TPU-coated yarns.

Figure 5 shows the results of the TPU-coated yarns’ electromechanical testing. Similar to the yarn characterizations presented above, the electrical signal follows the mechanical input. However, secondary peaks at the strain minima are visible. These secondary peaks are not as grave as they are for the base yarn but they are still worse than the HCB or HC40 versions.

As is apparent in Table 6, the yarn is stiffer in comparison with the other three due to the thick TPU coating. Although TPU has a relatively low stiffness, the modulus still increases around 50%. Furthermore, the modulus is nearly constant for the varying strain levels, indicating that the coating homogenizes the strain stress response by restricting fiber-to-fiber movement at the beginning, which leads to more overlap between structural and material deformation. This is supported by the fact that the gauge factor increases less with the rising strain level. The initial gauge factor is higher than it is for the regular HCB version but the final one is lower. Similarly, the amount of drift is also more stable over the varying strain levels.

However, the performance of the coated version is generally similar or worse in comparison with HCB and HC40. Consequently, due to the difficulties in processing, it is advisable to use the TPU-coated version only if either mechanical or electrical shielding from the environment is necessary. Moreover, it is possible to use the TPU-coated version as an electrode in capacitive-type strain sensors [21]. When comparing all four yarns, it has to be noted that, as expected, the relative resistance change or gauge factor is similar for all yarns. However, due to the different base resistivity of the S117 yarn, the absolute change is drastically higher for the S117 yarn. 

As described at the beginning of the paper, the electromechanical characterization of electrically conductive yarns is of course not at an end in itself. Based on this knowledge, it is possible to develop fully textile sensor structures. 

## 4. Conclusions

It can be concluded that silver-plated yarns are a suitable option for motion tracking. However, their properties vary drastically depending on textile parameters and integration processes. Therefore, a careful selection of the yarn type depending on the application is necessary. In order to be able to make such a profound decision, precise knowledge of the yarn properties is necessary. Therefore, a comprehensive characterization of electrically conductive textile materials is essential and thus was carried out here. 

One key result in our article was that the additional silver material of the HCB improves not only the conductivity but also the electromechanical stability of the yarn. The stability is further enhanced by the plying step, which is used to make the HC40 version. Consequently, the additional steps and required costs have merit for applications where this stability is required. However, even the HC40 yarns showed a considerable drift.

The analyses have also shown that there is still a lot of potential for the further development of existing conductive yarn materials. This concerns the electromechanical behavior as well as other properties, such as washing resistance, as has already been demonstrated in numerous previous publications. Depending on the intended application and the resulting requirements, a selection can be made from a growing number of commercial products or from further individual developments that must be pursued. 

Possible improvements should focus on the reduction in the drift and on the low sensitivity at low strain levels. In previous publications, this has been addressed by introducing a working range usually starting above a 5% strain. However, this is generally not practical for real-world applications, where a precise pre-strain of the textile or yarn is notoriously difficult to implement.

Approaches for optimizing or adapting the yarn properties can be, e.g., braiding or further coatings of the yarns. However, as the TPU-coated variant among the yarns examined already illustrates, improvements at one point are seldom not associated with disadvantages at another. Thus, the research field of electrically conductive yarns promises to continue providing exciting developments and not least because of the expected increase in demand for such materials.

## Figures and Tables

**Table 1:** Conductive yarns, which are investigated within this work.

Material Name	Abbreviation	Yarn Count (dtex)	Base Resistance (Ω/m)
Shieldex Multifilament 117/17 2-ply	S117	280	1500
Shieldex Multifilament 117/17 2-ply HCB	HCB	295	300
Shieldex Multifilament 117/17 2-ply HCB TPU	TPU	3000	300
Madeira HC 40	HC40	290	300

**Table 2:** Parameters of the testing regime.

Parameter	Samples per Yarn	Cycles per Strain Level	Strain Increments	Preload	Free Specimen Length	Testing Speed
Values	5	10	2%	0.5 cN/tex	250 mm	125 mm/min

**Table 3:** Metrics of the S117 yarn.

Strain Level (%)	4	6	8	10	12
Modulus	0.35	0.35	0.38	0.41	0.48
Gauge Factor	4.45	8.93	12.3	14.5	16.1
Drift (%)	63.5	46.5	42.4	42.7	44.2

**Table 4:** Metrics of the HCB yarn.

Strain Level (%)	4	6	8	10	12
Modulus	0.39	0.37	0.39	0.45	0.53
Gauge Factor	5.2	8.8	11.6	13.7	15.3
Drift (%)	38.9	26.3	25.2	25.8	27.7

**Table 5:** Metrics of the HC40 yarn.

Strain Level (%)	4	6	8	10	12
Modulus	0.39	0.38	0.40	0.44	0.52
Gauge Factor	8.7	14.7	18.6	21.0	22.3
Drift (%)	22.6	18.6	20.6	24.7	29.1

**Table 6:** Metrics of the TPU-coated yarn.

Strain Level (%)	4	6	8	10	12
Modulus	0.67	0.59	0.57	0.61	0.67
Gauge Factor	6.4	8.2	9.7	10.8	11.7
Drift (%)	32.2	31.5	27.3	27.4	27.7

## Data Availability

Data are available from the authors upon reasonable request.
